# Bound by Experience: Updating the Body Representation When Using Virtual Objects

**DOI:** 10.1177/00187208241258315

**Published:** 2024-06-14

**Authors:** Julia Eck, Roland Pfister

**Affiliations:** 1 9190 University of Würzburg, Germany; 2 26595 Trier University, Germany

**Keywords:** multisensory integration, motor control, perception-action, display-control or stimulus-response compatibility, perceptual-motor performance

## Abstract

**Objective:**

Four web-based experiments investigated flexibility of disembodiment of a virtual object that is no longer actively controlled. Emphasis was on possibilities to modify the timescale of this process.

**Background:**

Interactions with virtual objects are commonplace in settings like teleoperation, rehabilitation, and computer-aided design. These objects are quickly integrated into the operator’s body schema (embodiment). Less is known about how long such embodiment lasts. Understanding the dynamics of this process is crucial because different applied settings either profit from fast or slow disembodiment.

**Method:**

To induce embodiment, participants moved a 2D virtual hand through operating a computer mouse or touchpad. After initial embodiment, participants either stopped or continued moving for a fixed period of time. Embodiment ratings were collected continuously during each trial.

**Results:**

Results across all experiments indicated that embodiment for the virtual hand gradually increased during active use and gradually decreased after stopping to use it. Disembodiment unfolded nearly twice as fast as embodiment and showed a curved decay pattern. These dynamics remained unaffected by anticipation of active control that would be required in an upcoming task.

**Conclusion:**

The results highlight the importance of continuously experiencing active control in virtual interactions if aiming at inducing stable embodiment of a virtual object.

**Application:**

Our findings suggest that applications of virtual disembodiment such as virtual tools or interventions to affect a person’s body representation critically depend on continuous updating of sensorimotor experience. However, if switching between virtual objects, for example, during teleoperation or video gaming, after-effects are unlikely to affect performance.

## Introduction

Controlling an object can lead to embodiment, that is, it can lead to the object being perceived as if it were a part of one’s own body (Cardinali et al., 2009; Kalckert & Ehrsson, 2012; Maravita et al., 2003; Maravita & Iriki, 2004; Schettler et al., 2019). This is also true for virtual objects that are controlled by users for the purpose of executing an intended interaction in a digital workspace or environment (Schettler et al., 2019; Seinfeld et al., 2021). One applied example of interactions with virtual objects is teleoperation in industry (González et al., 2021) or health care (Diana & Marescaux, 2015). Many tasks that required a professional being physically present can now be performed by remote-controlled robots (Darvish et al., 2023; Moniruzzaman et al., 2022). Another example is the use of immersive or nonimmersive virtual reality technology for therapy and rehabilitation (Magrini et al., 2022; Zanatta et al., 2022). Examples include inducing sense of embodiment for an avatar with a healthy body-mass index for patients with anorexia nervosa (Keizer et al., 2016) or offering patients with motor impairments to play serious video games to regain motor control of immobilized limbs (Cuesta-Gómez et al., 2020; McNulty et al., 2015). Finally, interactions with virtual objects are also commonplace when playing video games for leisure, working with computer-aided design programs (Marion & Fixson, 2021), or simply using the mouse cursor to select a file on a standard desktop computer.

Integrating a virtual object into the body representation directly affects perception and action during the virtual interaction (Janczyk et al., 2013; Rubo & Gamer, 2019) and possibly even thereafter (Banakou et al., 2013; Reinhard et al., 2020). Such after-effects are sometimes desirable, for example, when aiming to alter a patient’s body representation in cognitive behavioral therapy of anorexia nervosa (Keizer et al., 2016) or in rehabilitation programs for motor impairment (Magrini et al., 2022; Zanatta et al., 2022). In other scenarios after-effects are not desirable, for example, when a surgeon needs to switch between different kinds of virtual objects during a teleoperation (Huang et al., 2021; Sengül et al., 2012). Therefore, it is pivotal to understand when and how previously embodied virtual objects are disembodied, including factors that either prevent or promote disembodiment. In the current study, we therefore investigated the temporal dynamics of both processes. As a model case, we chose the most common virtual interaction in terms of a simple computer mouse or touchpad controlling of the movement of a virtual object.

### Embodiment in Human–Computer Interaction

The embodiment of virtual objects has been studied in digital environments with various degree of realism ranging from simple nonimmersive computer mouse mediated interactions on a computer screen (Bergström et al., 2019; Kirsch et al., 2016) to controlling a realistic avatar in fully immersive virtual reality (Slater & Sanchez-Vives, 2014). These studies have in common that their theoretical rationale is rooted in multisensory integration models that conceive the body representation as resulting from binding of correlated cross-modal sensory signals (Ehrsson, 2020; Noel et al., 2018). Multisensory integration accounts predict that embodiment of an external object emerges whenever signals stemming from the object and signals stemming from one’s own body become integrated. A main factor promoting integration is experiencing temporal and spatial congruence between those signals, for example, visually perceiving movements of a virtual arm while kinaesthetically perceiving movements of one’s own arm at the same time (Liesner et al., 2021; Sanchez-Vives et al., 2010). However, there are also important differences between embodiment of virtual body parts or whole bodies in immersive virtual reality (Kilteni et al., 2013; Petkova & Ehrsson, 2008; Sanchez-Vives et al., 2010) and embodiment of less realistic virtual objects like a mouse cursor in nonimmersive settings (Bergström et al., 2019; Seinfeld et al., 2021). These differences pertain to different factors contributing to the sense of embodiment (Kilteni et al., 2012a).

Embodiment of virtual objects can comprise three different components (Gonzalez-Franco & Peck, 2018; Kilteni et al., 2012a). It can either refer to the experience that the virtual object feels like a part of one’s own body (body ownership), or the feeling that the self is being located within the virtual object (colocation), or the impression that movements of the virtual object are a direct consequence of one’s own intended actions (agency). For example, when controlling a humanoid avatar that is perceived from first-person perspective in immersive virtual reality, sense of embodiment for the avatar reflects the experience of all three components (e.g., Maselli & Slater, 2013). Whereas a sense of embodiment for less realistic objects during nonimmersive forms of human–computer interaction (HCI) mainly builds on agency experience, although agency in turn is supposed to promote changes in self-location and body ownership (Kirsch et al., 2016; Ma & Hommel, 2015b). The varying weight of the three aspects of embodiment relative to a current context is consistent with evidence suggesting a tight relationship between these components (Guy et al., 2022; Jeannerod, 2003; Kilteni et al., 2012a; Lin & Jörg, 2016; Ma & Hommel, 2015b; Maselli & Slater, 2013; Petkova et al., 2011). For the context of the current study, where we investigated subjective sense of embodiment for a 2D virtual hand that was controlled through movements of a computer mouse, interactions between the sense of agency and the other two components are particularly relevant.

Several studies showed how in the context of HCI agency experience might affect sense of ownership for the virtual object that is currently controlled. In one study, participants actively controlled a 2D virtual rectangle that was presented on a computer screen by means of a data glove that translated real hand movements to movements of the rectangle (Ma & Hommel, 2015b). Thus, movements of the real hand caused corresponding movements of the rectangle. Participants had to move the rectangle over the screen towards a virtual stick and received tactile stimulation of their hand if both virtual objects collided. In a control condition, participants were not allowed to move and passively received tactile stimulation of their hand while the virtual rectangle was touched by the virtual stick at the same time. Participants reported experiencing the virtual rectangle as a part of their body in the active condition but not in the passive condition. In another study, participants were immersed in a 3D virtual environment and either actively controlled (active condition) or only passively looked at (static condition) a virtual hand that was colocated with the real hand (Brugada-Ramentol et al., 2019). Participants reported experiencing a sense of ownership for the virtual hand in both conditions. However, when lowering anatomical plausibility of the virtual hand, ownership ratings decreased in the static condition but not in the active condition. Both findings suggest that embodiment of virtual objects particularly depends on agency during interactions with less realistic or nonhumanoid virtual objects.

Evidence from tool-embodiment studies suggests that agency experience might not only boost the sense of ownership but also the sense of self-location. Several studies have shown that active use of virtual or physical tools results in extension of peri-personal space—that is, the space that directly surrounds one’s own body—from the hand that holds the tool to the tip of the tool (Maravita et al., 2003; Schettler et al., 2019). The integration of a tool into one’s own peri-personal space is supposed to reflect sense of embodiment for the tool that is based on agency-driven changes in self-location (Kilteni et al., 2012a; Schettler et al., 2019). This kind of embodiment in the context of tool-use is usually not directly assessed through subjective ratings but indirectly inferred from behavioral indicators of peri-personal space extension, for example, performance in the cross-modal congruency task. In this paradigm, participants respond to tactile stimuli that are applied to a body part, for example, the hand, while ignoring visual stimuli that are presented at a spatially congruent or incongruent location relative to the tactile stimuli. Spatially incongruent visual stimuli interfere with task performance if presented near the hand and interference decreases with increasing distance between the visual stimulus and the hand. However, after some practice with a tool, task performance not only decreases for incongruent visual stimuli presented near the hand but also for incongruent visual stimuli presented near the functional part of the tool, which is taken as behavioral evidence for tool-embodiment (Maravita et al., 2003; Schettler, 2019).

One study adapted the cross-modal congruency task to the context of HCI to explicitly investigate whether a virtual object that is controlled by active movements of the user can become embodied in a similar way as tools become embodied after actively using them (Bergström et al., 2019). In this study, participants controlled a mouse cursor through moving a computer mouse or performing movements on a touchpad with their right hand. In a control condition, participants passively watched the mouse cursor moving over the screen. Tactile stimuli were applied at the right index finger or the right thumb, and visual stimuli were presented above or below the cursor on the screen. Visual stimuli presented above the cursor were assumed to be spatially congruent with tactile stimulation of the index finger and spatially incongruent with tactile stimulation of the thumb. For visual stimuli presented below the curser, the congruency mappings with respect to the stimulated finger was reversed. The participants’ task was to respond to tactile stimuli and to ignore the visual stimuli. Performance differences between congruent and incongruent trials were observed only if participants actively controlled the cursor. This result suggests that a mouse cursor can be integrated into the body representation if active control is experienced over it during a task.

Results from a related study suggest that embodiment of a mouse cursor can occur even without current experience of active control (Bassolino et al., 2010). In this study, users integrated the mouse cursor into the body representation if they controlled it through moving a computer mouse with their right or left hand. However, if participants only placed their hand on the computer mouse, without using it to control the cursor, embodiment of the mouse cursor occurred for the right hand, but not for the left hand. The authors interpreted their results in terms of agency experience (Bassolino et al., 2010; Serino et al., 2007). While the participants had a history of controlling the mouse cursor through moving the computer mouse with their right hand, they rarely used their left hand to operate the computer mouse in the past. Thus, remembered experience of agency with the computer mouse was only available for the right hand and not for the left hand which is why in the passive condition embodiment of the mouse cursor occurred only for the right and not for the left hand.

The distinctive role of agency for embodiment of virtual objects in nonimmersive HCI settings can be explained when construing bodily self-perception as causal inference (Ernst & Bülthoff, 2004; Kilteni et al., 2015; Samad et al., 2015). According to such models, the cognitive system infers a common cause for currently perceived correlated signals coming from different modalities. Whether incoming cross-modal signals become integrated or not depends on how closely they match in time and space, and it also depends on how plausible a common cause is, based on prior knowledge about one’s own body. For example, when seeing a realistic virtual body that is colocated with one’s own body in immersive virtual reality, perceived temporal and spatial congruency between visual signals coming from the avatar and proprioceptive signals coming from one’s own body would highly suggest a common cause for the current sensory experience. The same holds true for prior knowledge (the object looks like a body and its location is anatomically plausible), resulting in multisensory integration and embodiment of the virtual body (Kilteni et al., 2015; Noel et al., 2018; Samad et al., 2015). By contrast, in nonimmersive HCI, where the controlled virtual object is not colocated with one’s own body and possibly even does not look like a human body (part), low spatial correlation between visual and proprioceptive signals and prior knowledge would suggest that a common cause for the perceived temporally correlated visual and proprioceptive signals is unlikely. Additional information is therefore necessary to promote multisensory integration and thus embodiment. The experience of agency possibly compensates for the relatively low anatomical plausibility and the lack of colocation that is inherent to nonimmersive HCI settings (Braun et al., 2018; de Vignemont, 2010; Ma & Hommel, 2015a, 2015b; Samad et al., 2015). The current study leveraged these findings by using agency to elicit embodiment for a 2D virtual hand that could be actively controlled trough movements of a computer mouse or on a touchpad.

Similar to the procedure reported in the Bergström et al.’s (2019) study, participants in the current study controlled a mouse cursor through movements of the computer mouse or through operating a touchpad. Thus, active real hand movements immediately triggered corresponding movements of the virtual object. Our assumption that this procedure induced a sense of agency for the virtual object is based on evidence showing that experiencing control over one’s own movements and effects of these movements is supposed to induce agency (Braun et al., 2018; Haggard, 2017; Liesner et al., 2020; Ma & Hommel, 2015a, 2015b). In the Bergström et al.’s (2019) study, embodiment of the mouse cursor was indicated by performance in the cross-modal congruency task reflecting changes in self-location. In the current study, based on findings showing body ownership for controllable nonhumanoid 2D virtual objects (Ma & Hommel, 2015a, 2015b), embodiment of the virtual object was indicated through subjective reports of body ownership—or body part ownership, because we presented a virtual hand and not a whole virtual body. Therefore, in the reported experiments the sense of embodiment was operationalized as body part ownership that was based on agency. However, in the current study we were less interested in the mechanisms contributing to embodiment of virtual objects but rather aimed at investigating how initially induced ownership for a virtual object evolves if the task does no longer involve controlling of the virtual object.

To sum up, evidence suggests that not only complex 3D avatars (e.g., Maselli & Slater, 2013) but also simple 2D virtual objects (e.g., Bergström et al., 2019; Ma & Hommel, 2015a, 2015b) that are controlled through movements of a computer mouse or on a touchpad can become part of the body representation due to current or remembered experience of agency for the input device (e.g., computer mouse) and its digital counterpart (e.g., mouse cursor). However, the present literature comes with a sizeable gap on what happens to body representations after initial embodiment, because previous work has only just begun to assess disembodiment as a process (Abdulkarim et al., 2021; Eck et al., 2022; Pfister et al., 2021). Many critical questions, therefore, await answers from empirical studies. The present study aimed at extending the scarce literature on disembodiment through addressing the following two issues: how long does a previously embodied virtual object remain embodied after active control of the virtual object stops, and how flexibly can the body representation adapt to changing task demands? These questions are especially relevant for the context of HCI where task requirements, input devices, and visual representation of the users’ actions on the screen change quickly and dynamically.

### The Present Study

The current experiments followed the general design of a previous study (Eck et al., 2022). Participants controlled a 2D virtual hand through movements of a computer mouse or on a touchpad. Based on findings suggesting that humanoid appearance of external objects is positively related to body ownership (Lin & Jörg, 2016; Petkova & Ehrsson, 2008; Tsakiris et al., 2010), we choose to present a digital image of a realistic hand as the mouse cursor instead of an arbitrary virtual object (Bergström et al., 2019; Ma & Hommel, 2015a, 2015b). Each trial comprised an embodiment phase and a disembodiment phase. We use the terms “embodiment phase” and “disembodiment phase” to indicate the intended role of both phases for the experiment: The procedure during the embodiment phase aims at evoking feelings of embodiment for the virtual object, whereas the disembodiment phase allows for testing how disembodiment of the previously embodied virtual object unfolds. Because the current experiments aimed at replicating and extending earlier work from our lab (Eck et al., 2022; Pfister et al., 2021), for reasons of clarity and better comparability between studies, the terminology that was used in the current study for phases and conditions was mostly consistent with labels used in the preceding studies. During the embodiment phase participants had to continuously move the virtual hand back-and-forth from a target on the left side of the screen to a target on the right side of the screen, and vice versa. During the disembodiment phase participants either continued moving back-and-forth like before (active condition) or they stopped moving and passively watched the static virtual hand for the rest of the trial (no-movement condition). We measured embodiment and disembodiment of the virtual hand by asking participants several times in each phase to rate to which extent they experienced the virtual hand on the screen as a part of their body.

We found in the previous study that embodiment ratings continuously increased during the embodiment phase in both conditions (Eck et al., 2022). In the disembodiment phase of the active condition, ratings remained continuously high while ratings instantly dropped to preembodiment level as soon as participants stopped moving in the no-movement condition. The gradual increase of ratings during the embodiment phase was in line with our hypothesis and replicated previous observations of embodiment for 2D virtual objects that can be controlled through active movements (Bergström et al., 2019; Liesner et al., 2020; Ma & Hommel, 2015a, 2015b). However, the seemingly instant disembodiment of the virtual hand after stopping to move was unexpected. Based on previous work with physical rubber hands, we had predicted gradual disembodiment when the embodied virtual hand would no longer be updated through correlated visuo-motor signals (Abdulkarim et al., 2021; Pfister et al., 2021).

One possibility to account for the diverging findings for disembodiment in physical and virtual interactions is to assume that human agents might have learned (and therefore are prepared) to update body representations swiftly when interacting with computer technology (Eck et al., 2022). This could lead to instant disembodiment whenever an embodied virtual entity is no longer relevant for the task at hand. Alternatively, disembodiment of embodied virtual entities might still occur in a gradual fashion but on a faster timescale as compared to physical entities. Our previous study might not have been able to detect a gradual pattern for the simple technical reason that the interval between stopping to move and the first rating of the disembodiment phase was too long (20 seconds). Another methodological problem that might be responsible for the unexpected finding concerns our rating scale. In the previous study, participants could indicate their sense of embodiment for the virtual hand on a scale consisting of nine categories that were represented by numbers ranging from 1 to 9. Semantic anchors were provided for the rating scale as follows: A rating of 1 indicated no embodiment as reflected in the corresponding semantic anchor: “I feel no relation between the hand on the screen and my body.” Moderate sense of embodiment was suggested by another semantic anchor at a rating of 3: “I could imagine the hand on the screen as an extension of my body.” A rating of 7 represented the statement: “I have the feeling that the hand on the screen is a part of my body,” suggesting solid embodiment. Finally, rating 9 indicated particularly strong embodiment as was implied by the relevant semantic anchor: “I have the feeling that the hand on the screen is my own hand.” These semantic anchors might have possibly biased the responses of the participants.

In the current study, we ruled out the methodological concerns of the previous study and tested the assumption that anticipating task demands of an upcoming task might speed up or slow down the disembodiment of virtual objects. In Experiment 1 the interrating intervals for the disembodiment phase were 10 seconds, that is, half as long as in the previous study. Shortening the interrating intervals allowed us to test whether disembodiment would already be completed within an even shorter timeframe, as would be predicted by instant disembodiment, or whether ratings would decrease more gradually during this time. Based on the previous study we expected instant disembodiment of the virtual hand shortly after participants would stop moving. In Experiment 2, we aimed at validating the previous results by using a rating scale without any semantical anchors. Finally, in Experiment 3 and Experiment 4 we tested whether the disembodiment of the virtual hand can be prevented if users are explicitly informed that after a brief movement break, they would have to actively control the virtual hand again. Based on findings showing that recalled or imagined agency can enhance embodiment of an external object (Bassolino et al., 2010; Cardinali et al., 2021; Liepelt et al., 2017), we assumed that anticipated agency might prevent disembodiment.

The four experiments that we report in the following were online studies and participants conducted the experiments remotely on their own personal computers. Participants were prompted to use a desktop computer for the study and to refrain from using any touchscreen functionality of their device. At the end of the study, participants were invited to report their idea of what the purpose of the current study was. We checked whether including datasets of participants whose description came close to the actual aim of the study by mentioning concepts such as body representation or embodiment (none in Experiment 1; three in Experiment 2; seven in Experiment 3; sixteen in Experiment 4) would change the observed pattern of results. This was not the case, however. All experiments were conducted according to the ethical regulations of the Ethics Committee of the Institute of Psychology, University of Würzburg. Informed consent was obtained from each participant.

## Experiment 1

The main aim of Experiment 1 was to test whether an embodied virtual hand would become disembodied immediately after stopping to move, or whether disembodiment would occur more gradually over time. We therefore modified a previous design (Eck et al., 2022) to yield improved temporal resolution specifically during disembodiment.

### Methods

#### Participants

For Experiment 1, 40 participants were recruited on the online platform Prolific (https://www.prolific.co/). This sample size was suggested by a power analysis based on previous data (Eck et al., 2022). The current sample sizes came with a power of 1-β > 99% to detect the instant disembodiment that we observed in Experiment 1 of the previous study (comparison of the last rating before the disembodiment phase and the first rating of the disembodiment phase of the no-movement condition; *d*_*z*_ = 0.84). Due to a programming error, two datasets were lost, whereas two additional datasets had to be discarded due to our preregistered exclusion criteria (see *Data Processing* section for more details). The final sample of 36 participants still provided a power for 1-β > 99% if assuming the effect size of the previous study. The mean age of the participants was 23.50 years (range: 18–44). Most of the participants (16 female, 17 male, and three did not specify their gender) described themselves as right-handed (30), two participants indicated being left-handed, and four participants did not report their handedness. According to self-disclosure, 13 participants controlled the virtual hand through movements of a computer mouse, while 14 participants operated a touchpad, one participant used a trackpad, and eight participants did not disclose which input device they had used for the study.

#### Apparatus and Stimuli

The experiment was programmed in PsychoPy (Version v2020.2.8). Computer programs and stimulus material for all reported experiments are available online (https://osf.io/48tfy/?view_only=03bf5910074c4426b7989f769ecdad8f).

**Figure 1.**
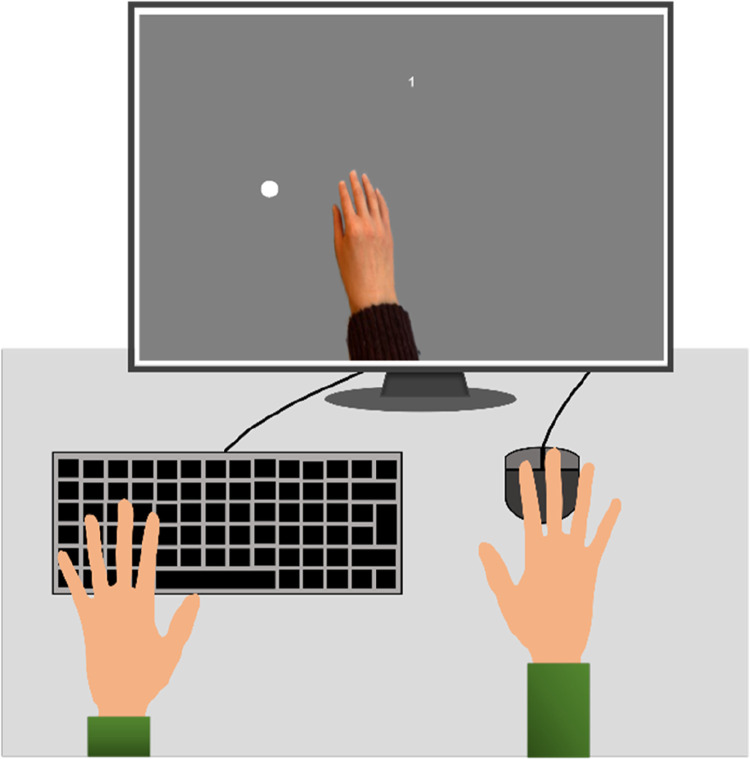
Main setup and stimuli of the current web-based study.

At the beginning of the experiment, an image of a hand appeared at the center of the computer screen and extended to the screen’s bottom edge. The hand’s posture was chosen to resemble a real hand that is resting on a computer mouse or on a touchpad. Movements along the x-coordinate of the mouse cursor caused left or right movements of the virtual hand. Movements along the y-coordinate had no effect on the position of the virtual hand. Two circular targets were presented either at the left or right side of the screen opposite to each other. The position of each target was chosen in such a way that when reaching one of the targets it was possible to directly point with the virtual index finger at this target. The rating question and other text stimuli, like instructions or feedback, were presented right above the virtual hand. Figure 1 illustrates an exemplary setup and the presentation of the main stimuli.

#### Procedure

**Figure 2.**
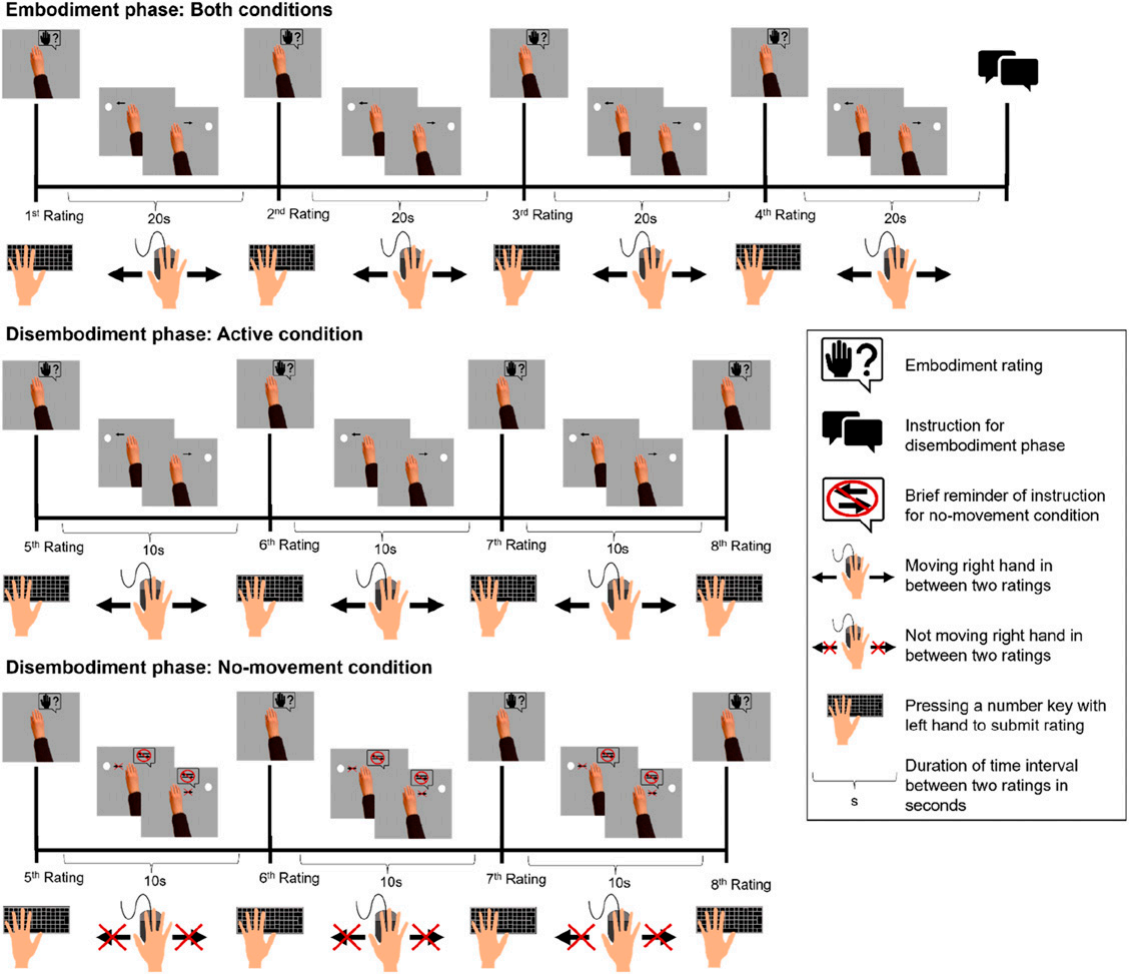
Events of one trial of the active and the no-movement condition for Experiment 1: Events on the computer screen are depicted above the timeline and corresponding actions of the participants below the timeline. *Note.* In phases without movement participants performed an attention task and the short question to test performance in the attention task was presented right before the respective rating question (for more details on the attention task please see the section *Procedure* for Experiment 1).

Figure 2 illustrates the events of one trial that entailed an embodiment phase and a disembodiment phase. The procedure for the embodiment phase was the same in every trial whereas the procedure of the disembodiment phase differed between conditions. At the beginning of each trial, the virtual hand was presented at the center of the computer screen and participants were asked to rate (1^st^ rating) to which extent they experience the hand on the screen as a part of their body. For Experiment 1 we used the rating scale from our previous study (Eck et al., 2022). The exact wording of the rating question was “Does the hand on the screen feel like a part of your body?” The rating scale ranged from 1 to 9. Semantic anchors were provided at the rating positions 1 (“I feel no relation between the hand on the screen and my body”), 3 (“I could imagine the hand on the screen as an extension of my body”), 7 (“I have the feeling that the hand on the screen is a part of my body”), and 9 (“I have the feeling that the hand on the screen is my own hand”). To submit a rating, participants had to press a number key on the keyboard. Participants were free to enter any number between 1 and 9 which best represented their current sense of embodiment. After entering the rating, participants started moving the virtual hand back-and-forth between two circular targets. Operating the virtual hand was realized through corresponding movements of the computer mouse or on the touchpad (embodiment phase). Only one target was visible at a time. Reaching the current target made this target disappear while the opposite target appeared on screen. To measure the progression of embodiment, participants were asked to submit embodiment ratings every 20 s (2^nd^–5^th^ rating). The embodiment phase in the current study lasted approximately 80 s. The length of the induction phase was based on previous work indicating the rubber hand illusion to occur within about 1 minute (Kalckert & Ehrsson, 2017) and on studies investigating embodiment of 2D virtual objects in a nonimmersive HCI (Ma & Hommel, 2015a, 2015b).

Five seconds before participants had to submit their last rating for the embodiment phase they received a brief instruction for the disembodiment phase, which started right after the rating. For the no-movement condition, participants were instructed to stop moving and to hold their hand still. For the active condition participants continued moving the virtual hand like they had done during the embodiment phase before. During the disembodiment phase, we asked participants every 10 seconds to rate embodiment for the virtual hand (6^th^–8^th^ rating). The interrating intervals were half as long as in the previous study (Eck et al., 2022). The shorter interrating intervals for the disembodiment phase of Experiment 1 aimed at testing whether the interrating intervals of the previous study (20 s) were too long to detect a gradual decrease of embodiment ratings that, in the previous study, might have been completed already before submission of the first rating of the disembodiment. The disembodiment phase stopped after the third rating (overall duration approx. 30 s). We assumed that a longer disembodiment phase would not be necessary because in the previous study we found that embodiment ratings dropped within the first 20 s after participants stopped moving and did not decrease further thereafter.

During the embodiment phase and the active condition of the disembodiment phase participants received warnings if they moved too fast (>20 back-and-forth movement during embodiment phase or >8 back-and-forth movements during the disembodiment phase; trials were excluded if participants exceeded these thresholds by 5 or more movements). During the no-movement condition of the disembodiment phase, participants received a warning if they moved and a brief reminder of the instruction for the no-movement condition was presented above the virtual hand in between the ratings. In addition, we had a simple attention task during the no-movement condition of the disembodiment phase. For this attention task the circular targets appeared or disappeared alternatingly every second. It was randomly assigned whether an interrating interval lasted 9, 10, or 11 seconds and participants were instructed to count how many targets appeared. After each embodiment rating, we asked participants to report how many targets they had counted during the last interrating interval. With the attention task we aimed to control that participants kept attending the virtual hand after stopping to move. Participants were instructed to operate the computer mouse or the touchpad with the right hand and to enter numbers for the embodiment ratings or the attention task with their left hand.

Participants were assigned to each condition in a full within-subject design. Conditions alternated across trials, with the initial condition being determined randomly. We had eight trials in total, so that each condition was completed four times. Participants were able to take short breaks between trials. They were encouraged to take their hand off the computer mouse or touchpad and to relax for several minutes during these breaks. After completing the last trial participants were asked to enter demographic data and to report their personal assumptions about the aim of the current study.

#### Data Processing

First, we filtered the data according to the preregistered criteria for trial exclusion (https://aspredicted.org/i58va.pdf). The scale that we provided for the embodiment ratings ranged from 1 to 9 and we excluded all trials with an embodiment rating lower than 3 at the end of the embodiment phase (4^th^ rating position). A rating of 3 represented the statement “I could imagine the hand on the screen as an extension of my body.” Ratings lower than 3 indicate that participants did not affirm this statement suggesting that no embodiment had emerged for the virtual hand. Such cut-off criteria in body ownership illusions aim at separating participants who respond to the embodiment induction from nonresponders (e.g., Kalckert & Ehrsson, 2017). Further trials were excluded if participants moved too fast in phases where they were instructed to move (more than 25 back-and-forth movements during an interrating interval of the embodiment phase or more than 13 back-and-forth movements during an interrating interval in the disembodiment phase of the active condition) or if they moved more than three times in phases where movement was not allowed. Finally, poor performance in the attention task during the disembodiment phase also led to trial exclusion (two or more mistakes). Only data from participants with at least one valid trial per condition were included in the statistical analysis. For the final sample of 36 participants, data processing resulted in exclusion of 16.7 % of the trials.

#### Data Analysis

All analyses reported below were performed in R (version 4.2.1). We used Bayesian statistics that were computed with the R package BayesFactor (version 0.9.12–4.4) to test predicted equality between conditions. The null hypothesis of equality was accepted if BF_01_ > 3.0. For computing the Bayes Factor (BF) for the current study, evidence for the null hypothesis that sample means are comparable between rating positions or conditions, were divided by evidence for the alternative hypothesis that sample means differ between rating positions or conditions. The subscript numbers next to the letters BF represent the position of the respective model within the BF ratio, that is, in BF_01_ the first subscript number, 0, indicates that the null hypothesis model was the numerator of the BF ratio and the second number, 1, indicates that the alternative hypothesis model was the denominator. Therefore, BF_01_ > 1 would suggest that evidence for the null hypothesis is higher than for the alternative hypothesis and the vice versa would be true for BF_01_ < 1. Commonly, a BF_01_ that is >1 and <3 is interpreted as anecdotal evidence, 3 < BF_01_ < 10 as moderate evidence and BF_01_ > 10 as strong evidence for the statistical model that is represented by the numerator of the Bayes factor fraction (Keysers et al., 2020). We used classical frequentist statistics to test for predicted differences between conditions or rating positions. Differences with a *p*-value <0.05 were considered significant. Raw data and syntax files for recreating the analyses for all reported experiments are available online (https://osf.io/48tfy/?view_only=03bf5910074c4426b7989f769ecdad8f).

### Results

**Figure 3.**
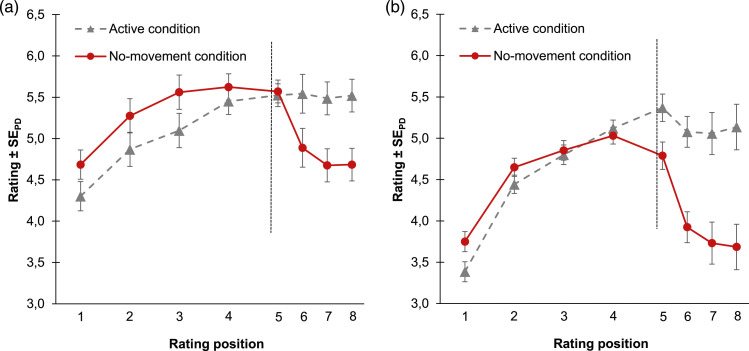
Mean embodiment ratings for Experiment 1 (a) and Experiment 2 (b) as a function of rating position and condition. *Note*. The vertical line marks the timepoint when the embodiment phase ended and the instruction for the disembodiment phase was presented (right before the 5^th^ rating). During the disembodiment phase interrating intervals were half as long as during the embodiment phase. Error bars indicate +/−1 standard error of paired differences between the active condition and the no-movement condition (Pfister & Janczyk, 2013).

Figure 3(a) shows the mean embodiment ratings as a function of rating position (1^st^–8^th^) and condition (active vs. no-movement).

#### Embodiment phase

The predicted increase of embodiment ratings was tested with a 2 × 2 analysis of variance (ANOVA) with the factors rating position (1^st^ vs. 4^th^ rating) and condition (active vs. no-movement). The ANOVA showed a significant main effect of rating position, *F* (1, 35) = 26.59, *p* < .001, η_p_^2^ = .43. The main effect of condition, *F* (1, 35) = 2.84, *p* = .101, η_p_^2^ = .07, and the interaction, *F* (1, 35) < 1, were not significant. A first Bayesian *t* test compared mean embodiment ratings between conditions across all rating positions of the embodiment phase. The result did not support the null hypothesis that rating levels were comparable between conditions, BF_01_ = 0.06. The second Bayesian *t* test compared the means of embodiment ratings between conditions specifically for the 4^th^ rating position. The result provided evidence for the null hypothesis of equality, BF_01_ = 3.76. To sum up, the results of the Bayesian statistics suggest that the average levels of embodiment were different between conditions whereas embodiment ratings at the 4^th^ rating position were comparably high in both conditions.

#### Disembodiment Phase

To test whether embodiment ratings decreased in the no-movement condition relative to the stable rating levels in the active condition we also conducted a 2 (condition: active vs. no-movement) x 2 (rating position) ANOVA for the disembodiment phase. As for rating positions, we now compared the rating right before the instruction (4^th^ rating position) with the last rating of the disembodiment phase (8^th^ rating position). The ANOVA revealed a significant main effect of condition, *F* (1, 35) = 5.71, *p* = .022, η_p_^2^ = .14 and a significant main effect of rating position, *F* (1, 35) = 11.93, *p* = .001, η_p_^2^ = .25. The interaction of both factors was also significant, *F* (1, 35) = 15.78, *p* < .001, η_p_^2^ = .31.

In addition to the ANOVA, we performed a series of follow-up *t*-tests and Bayesian *t*-tests to explore the dynamics of disembodiment in each condition. A Bayesian *t* test supported the null hypothesis that ratings are similar for the comparison between the 4^th^ and 8^th^ rating position for the active condition, BF_01_ = 5.44. We further computed *t*-tests to compare the 4^th^ and 5^th^ rating as well as the 5^th^ and 6^th^ rating for the no-movement condition. While the first contrast was not significant, *t* (35) = 0.59, *p* = .557, *d*_*z*_ = 0.10, ratings decreased significantly between the 5^th^ and 6^th^ rating position, *t* (35) = 3.23, *p* = .003, *d*_*z*_ = 0.54. The result of a Bayesian *t* test, which compared the 6^th^ and 8^th^ rating for the no-movement condition did not return evidence for the null hypothesis that ratings between both rating position are comparable, BF_01_ = 1.21. For the attention task participants responded in 85.03% of trials correctly.

### Discussion

The results suggest that the virtual hand became gradually embodied during the embodiment phase while participants moved it across the computer screen. If participants continued moving the virtual hand after initial embodiment ratings remained continuously high. However, if participants stopped moving, embodiment ratings decreased rapidly during the initial part of the disembodiment phase. The main decrease occurred during the first 10 seconds after onset of the no-movement condition and ratings slightly decreased further during the subsequent interrating interval. Thus, like in our previous study (Eck et al., 2022), we again observed that embodiment ratings gradually increased over the course of the embodiment phase and decreased about twice as fast during the disembodiment phase of the no-movement condition. The better temporal resolution of the present design, however, showed that embodiment ratings did not drop instantly after stopping to move but decreased more gradually over time. This gradual decrease followed a curved decay function with most but not all of the decrease occurring during the first 10 seconds of the disembodiment phase. (Had we observed complete disembodiment already for the first rating of the disembodiment phase, we would have had to adjust the design to use even shorter interrating intervals to gauge for actually immediate disembodiment. These adjustments were not necessary based on the data pattern emerging from Experiment 1, however).

## Experiment 2

Experiment 2 aimed at validating the results of Experiment 1 by using a more common rating scale. More precisely, in Experiment 2 we wanted to rule out that the results of Experiment 1 might have been biased by the semantic anchors that were provided for the rating scale. To this end we removed all semantic anchors and slightly changed the rating format based on rating scales that were used in several other studies that investigated the embodiment of external objects (e.g., Keizer et al., 2016; Kokkinara & Slater, 2014; Sanchez-Vives et al., 2010). We expected to replicate the results of Experiment 1.

### Methods

#### Participants

We collected Data from 40 undergraduates, recruited at the University of Wuerzburg through the online platform SONA (https://psywue.sona-systems.com). Participants received course credit for participation.

Data preprocessing followed the same procedure as in Experiment 1 (preregistration for Experiment 2: https://aspredicted.org/m4jb5.pdf) and we analyzed only data from participants with at least one valid trial per condition. According to this procedure we had to exclude eight datasets entirely and 13.3% of trials for the datasets of the remaining 32 participants. The participants (five male, 27 female) were on average 21.44 years old (range: 18–37). Nearly all participants (29) referred to their right hand as the dominant hand and three participants reported that the left hand was their dominant hand. Participants reported more frequently using a touchpad for the study (18 participants) than using a computer mouse (14 participants).

#### Stimuli, Apparatus, and Procedure

Compared to Experiment 1, the only procedural change concerned the rating scale. Instead of the rating scale with the semantic anchors we now asked participants to indicate to which extent they agree or disagree (0 = totally disagree; 9 = totally agree) with the following statement: “I feel as if the hand on the screen is my hand.” (wording in German: “Ich habe das Gefühl, als wäre die Hand am Bildschirm meine eigene Hand.”; all instructions of Experiment 2 were translated to German because we used the local participant pool of a German university to recruit our participants). As for Experiment 1, for Experiment 2 we also relied on a within subject design.

### Results

Figure 3(b) shows mean embodiment ratings for both conditions. Analyses were as for Experiment 1.

#### Embodiment Phase

The main effect of rating position was significant, *F* (1, 31) = 39.90, *p* < .001, η_p_^2^ = .56, as was the interaction, *F* (1, 31) = 14.92, *p* = .001, η_p_^2^ = .32. The main effect of condition was not significant, *F* (1, 31) = 2.11, *p* = .156, η_p_^2^ = .06. Results of Bayesian *t*-tests support the hypothesis that ratings were comparably high between conditions across all rating positions, BF_01_ = 11.23, and also when comparing ratings between conditions only for the 4^th^ rating position, BF_01_ = 3.70.

#### Disembodiment Phase

There was a significant main effect of condition, *F* (1, 31) = 29.50, *p* < .001, η_p_^2^ = .49, and a significant main effect of rating position, *F* (1, 31) = 13.92, *p* = .001, η_p_^2^ = .31. Both factors interacted significantly, *F* (1, 31) = 20.29, *p* < .001, η_p_^2^ = .40. The result of a Bayesian *t* test comparing the embodiment ratings at the 4^th^ and 8^th^ rating positions for the active condition, BF_01_ = 5.25, provided evidence for the null hypothesis of equality, suggesting that ratings were comparably high at both rating positions of the active condition. Ratings did not differ between the 4^th^ and 5^th^ rating position of the no-movement condition, *t* (31) = 1.69, *p* = .101, *d*_*z*_ = 0.30, but there was a significant difference between the 5^th^ and 6^th^ rating position of the no-movement condition, *t* (31) = 4.40, *p* < .001, *d*_*z*_ = 0.78. For the comparison of the first (6^th^ rating position) and the last (8^th^ rating position) rating of the disembodiment phase of the no-movement condition, a Bayesian *t* test did not yield evidence for the null hypothesis of equality between both rating positions, BF_01_ = 1.41. During the attention task participants responded correctly in 87.58 % of trials.

### Discussion

Embodiment ratings gradually increased during the embodiment phase. During the disembodiment phase of the active condition ratings remained constantly high at the level of the last rating prior to the instruction for the disembodiment phase. During the initial part of the disembodiment phase of the no-movement condition ratings decreased rapidly. Subsequently, ratings continued decreasing but considerably slower than at the beginning of the disembodiment phase. Experiment 2, therefore, replicated the main results of Experiment 1 for the disembodiment phase despite the changed rating scale. The replication suggests that the semantic anchors used in Experiment 1 and in our previous Study (Eck et al., 2022) did not bias the obtained results.

## Experiment 3

In Experiment 1 and Experiment 2 we had seen that, after stopping, the virtual hand was disembodied nearly twice as fast as it was previously embodied. Speculatively, the disembodiment of the virtual hand was particularly fast because participants might have been prepared to disembody the virtual hand through anticipation of the task requirements of the disembodiment phase of the no-movement condition. Such anticipation effects might have been based on the instructions for the disembodiment phase of the no-movement condition (de Vignemont, 2010; Kirsch, 2021; Ten Oever et al., 2016). In Experiment 3 we tested this hypothesis in a new condition, the stop-and-go condition. In the stop-and-go condition, participants were explicitly instructed that after the embodiment phase, they would have to stop moving the virtual hand (stop-part) only for two interrating intervals (20 s), whereas they would resume moving back-and-forth thereafter (go-part). This procedure enabled testing whether participants anticipate task requirements of the go-part of the stop-and-go condition and adapt their body representation accordingly before starting to move again. Thus, in Experiment 3 we tested the hypothesis that ratings would decrease less after stopping to move when anticipating to resume the movement task shortly after as compared to a setting that instructed participants to stop moving completely (no-movement condition).

### Methods

#### Participants

For Experiment 3 we recruited 40 new participants on Prolific (https://www.prolific.co/). The criteria for trial exclusion were the same as in the previous experiments (preregistration for Experiment 3: https://aspredicted.org/i58va.pdf). Individual datasets were excluded from the data analysis if there was less than one valid trial in any condition.

Data preprocessing resulted in exclusion of five datasets and 17.9 % of trials for the remaining 35 datasets. Twelve participants indicated to be female and 23 male. They were between 18 and 48 years old with a mean age of 27.83 years. From the 34 participants who reported their handedness 29 were right-handed and five left-handed. While one participant did not report which device he or she used to complete the study, most of the participants (23) reported that they had controlled the virtual hand through operating a computer mouse, and the remaining participants (11) stated that they used a touchpad for the experiment.

#### Stimuli, Apparatus, and Procedure

**Figure 4.**
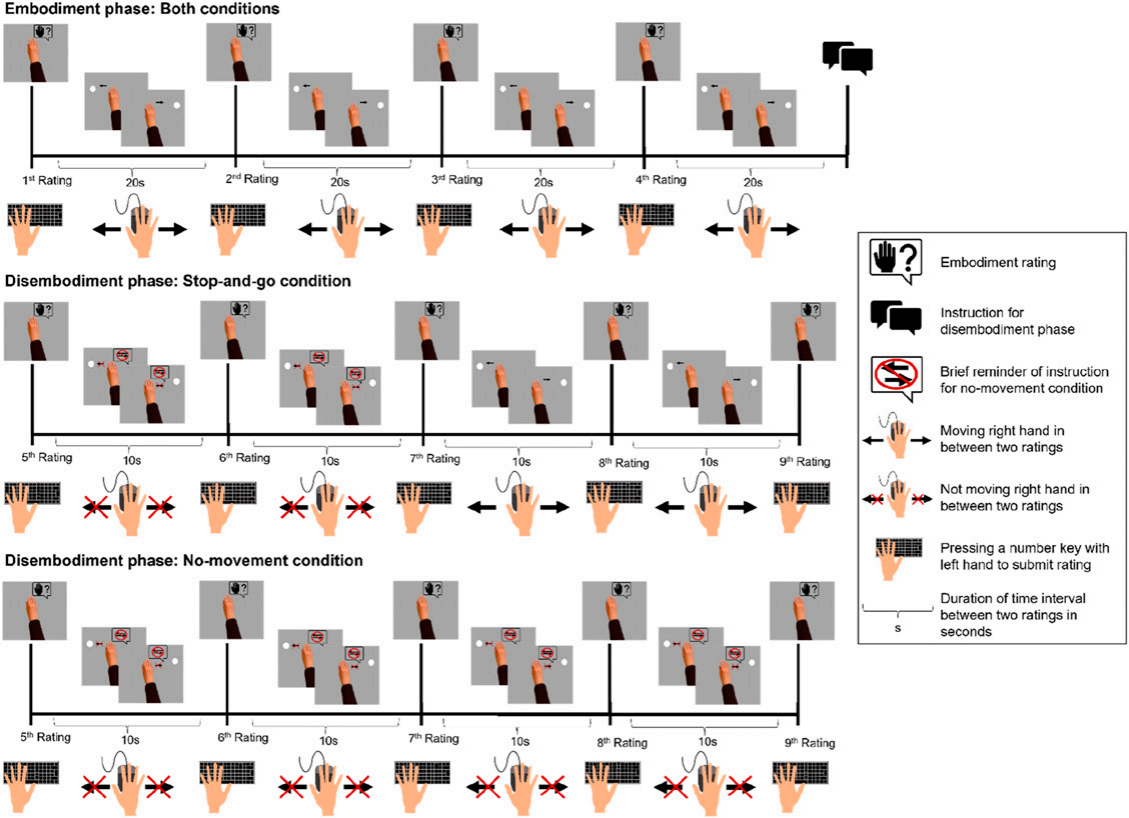
Events of one trial of the active and the no-movement condition for Experiment 3: Events on the computer screen are depicted above the timeline and corresponding actions of the participants below the timeline. *Note.* In phases without movement participants performed an attention task and the short question to test performance in the attention task was presented right before the respective rating question (for more details on the attention task please see the section *Procedure* for Experiment 1).

The experimental procedure of Experiment 3 is illustrated in Figure 4. The overall procedure for the embodiment phase and the disembodiment phase of the no-movement condition was the same as in the preceding experiments. However, an important change was that in Experiment 3 we replaced the active condition with a new condition, the stop-and-go condition. Like before, the embodiment phase was identical for both conditions and the critical manipulation was implemented in the disembodiment phase. While in the no-movement condition participants were supposed not to move for the whole duration of the disembodiment phase (now lasting 40 s), for the stop-and-go condition they were instructed to stop moving only for the duration of two successive rating questions. After participants rated embodiment for the virtual hand for the second time during the disembodiment phase of the stop-and-go condition they started moving again back-and-forth for the rest of the disembodiment phase (20 s). Hence, we had two parts in the stop-and-go condition—a no-movement part and a movement part. In both conditions, participants performed the same attention task as in the previous experiments during periods without movement. To be able to split the disembodiment phase in two parts of equal duration, we had four ratings during the disembodiment phase in Experiment 3 (6^th^ to 9^th^ rating position). For the embodiment ratings we used the same rating scale as in Experiment 1. Brief reminders of the critical instruction were presented during the experiment in both conditions. Like in the previous experiments, each participant went through each condition.

### Results

**Figure 5.**
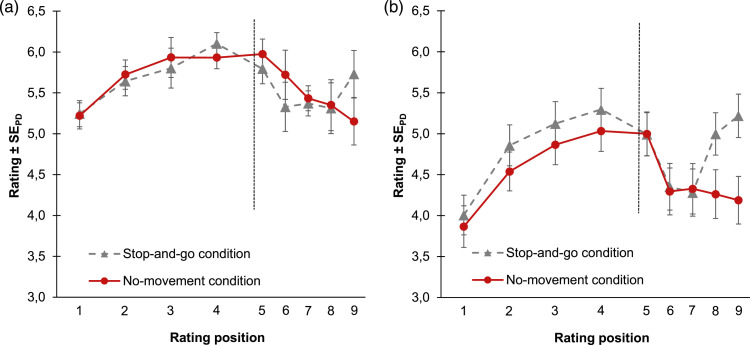
Mean embodiment ratings for Experiment 3 (a) and Experiment 4 (b) as a function of rating position and condition. *Note*: The vertical line marks the timepoint when the embodiment phase ended and the instruction for the disembodiment phase was presented (right before the 5^th^ rating). During the disembodiment phase interrating intervals were half as long as during the embodiment phase. Error bars indicate +/−1 standard error of paired differences between the no-movement condition and the stop-and-go condition (Pfister & Janczyk, 2013).

Figure 5(a) shows mean embodiment ratings as a function of rating position (1^st^–9^th^) and condition (no-movement vs. stop-and-go).

#### Embodiment Phase

A 2 × 2 ANOVA with the factors rating position (1^st^ vs. 4^th^ rating position) and condition (no-movement vs. stop-and-go condition) revealed a main effect of rating position, *F* (1, 34) = 20.24, *p* < .001, η_p_^2^ = .37. There was neither a main effect of condition, *F* (1, 34) < 1, nor a significant interaction, *F* (1, 34) < 1. A first Bayesian *t* test comparing embodiment ratings between condition across all rating positions indicated that the average embodiment ratings were comparable between conditions, BF_01_ = 10.63. In a second Bayesian *t* test we compared embodiment ratings between condition for the 4^th^ rating position. Although the result suggested that evidence for the null hypothesis is higher than for the alternative hypothesis, BF_01_ = 2.63, this evidence is anecdotal and therefore the null hypothesis, that ratings are comparably high between conditions, is not accepted.

#### Disembodiment Phase

We first tested whether during the initial part of the disembodiment phase ratings in the stop-and-go condition decreased less than in the no-movement condition by computing a 2 × 2 ANOVA with the factors rating position (5^th^ vs. 7^th^ rating) and condition (no-movement vs. stop-and-go condition). There was a nonsignificant trend for the main effect of rating position, *F* (1, 34) = 3.69, *p* = .063, η_p_^2^ = .10. The main effect of condition, *F* (1, 34) < 1, and the interaction were not significant, *F* (1, 34) < 1. For the factor rating position, we erroneously preregistered that we would compare ratings between rating position 5 and rating position 9. For testing the hypothesis of Experiment 3—namely, that ratings would decrease more slowly in the stop-and-go condition compared to the no-movement condition during the no-movement part (5^th^–7^th^ rating position) and would increase rapidly after participants resume moving or even slightly before—requires the comparison between rating position 5 and rating position 7.

We further explored the evolution of disembodiment in more detail by performing *t*-tests between several rating positions in each condition respectively. For the no-movement condition neither the *t* test comparing the 5^th^ and 6^th^ rating, *t* (34) = 1.36, *p* = .184, *d*_*z*_ = 0.23 nor the contrast between the 6^th^ and 7^th^ rating, *t* (34) = 1.34, *p* = .188, *d*_*z*_ = 0.23, were significant. These results do not support the predicted initial rapid decrease of ratings but instead suggest that ratings decreased steadily from the beginning of the disembodiment phase to the end. Because of this unexpected observation the preregistered Bayesian *t* test where we planned to test whether the levels of the rating after the initial decrease and the last rating of the disembodiment phase are equal was not performed.

For the stop-and-go condition the *t* test comparing the 5^th^ and 6^th^ rating revealed a nonsignificant trend towards a difference between both ratings, *t* (34) = 1.83, *p* = .076, *d*_*z*_ = 0.31. The contrast between the 6^th^ and 7^th^ rating was not significant, *t* (34) = −0.63, *p* = .530, *d*_*z*_ = −0.11. Further we tested whether embodiment was fully reinstated in the stop-and-go condition after participants started moving again by comparing the 5^th^ and 8^th^ rating as well as the 5^th^ and 9^th^ rating with Bayesian *t*-tests, respectively. The results of the first Bayesian *t* test provided higher evidence for the alternative hypothesis than for the null hypothesis, BF_01_ = 0.75, suggesting that ratings were different between the 5^th^ and 8^th^ rating position. The result of the second Bayesian *t* test, BF_01_ = 5.27, favored the null hypothesis over the alternative hypothesis suggesting that ratings were similar between the 5^th^ and 9th rating position. The proportion of trials where participants responded correctly to the attention check was 81.6 %.

### Discussion

In Experiment 3 we observed the expected gradual increase of ratings during the embodiment phase for both conditions. However, the results for the disembodiment phase do not support our hypothesis. In the no-movement condition, instead of the predicted rapid decrease at the beginning of the disembodiment phase ratings decreased continuously during the whole disembodiment phase. For the stop-and-go condition, we expected a less pronounced decrease of embodiment ratings during the initial part of the disembodiment phase than during the no-movement condition and a fast increase of embodiment ratings right before starting to move again or at least shortly thereafter. Challenging this hypothesis, the overall progression of disembodiment during the initial part of the disembodiment phase was comparable between conditions and the observed reinstatement of embodiment after participants resumed moving in the stop-and-go condition was descriptively slower than expected. Taken together, the results do not support the hypothesized anticipatory effects of task instruction on disembodiment. However, a possible alternative explanation for the unexpected findings could be that participants might have had difficulties to distinguish between conditions properly because both might have been represented as one and the same task entailing three main components: moving, stopping to move for a short while, resuming to move thereafter. Due to the repeated measures design of Experiment 3, participants might have perceived the expected movement-interruptions in both conditions as similar enough to fall into the same category. Therefore, both tasks might have triggered similar expectations and have been possibly performed in a similar manner.

## Experiment 4

In Experiment 4 we assessed whether the unexpected results of Experiment 3 may reflect carry-over effects from one condition to the other. For this in Experiment 4 we replicated Experiment 3 by varying conditions between participants instead of within participants.

### Methods

#### Participants

Participants were recruited via the online platform Prolific (https://www.prolific.co/). For the between-subjects design, a power analysis assuming a power of 80% and a medium effect size (*d*_*z*_ = 0.5) suggested a sample size of 64 participants per group. Therefore, we collected data from 128 new participants in total.

The criteria for trial exclusion were the same as in the previous experiments (preregistration for Experiment 4: https://aspredicted.org/vt8zv.pdf) and only datasets were analyzed that contained at least one valid trial per condition. Accordingly, we had to exclude datasets from 12 participants, and 15.7% of trials for the remaining 116 participants. Out of these participants, 56 were in the no-movement condition and 60 participants in the stop-and-go condition. For the whole sample, mean age was 29.80 years (range = 18–68). In the no-movement condition participants were on average 28.32 years old (SD = 8.85; range = 18–58) and 31.18 years (SD = 11.96; range = 18–68) in the stop-and-go condition. From the 110 participants who disclosed their gender 67 described themselves as male, 42 as female and one as nonbinary (no-movement condition: 34 male and 20 female participants; stop-and-go condition: 33 male and 22 female participants, one nonbinary participant). Three participants left no information concerning their handedness. From the remaining participants 103 reported to be right-handed and 10 left-handed (no-movement condition: 50 right- and five left-handed participants; stop-and-go condition: 53 right- and five left-handed participants). While 19 participants did not report which device they used for the study, most of the participants (68) stated that they used a computer mouse and the remaining participants (29) indicated that they participated using a touchpad (no-movement condition: 34 computer mouse and 12 touchpad users; stop-and-go condition: 34 computer mouse and 17 touchpad users).

#### Stimuli, Apparatus, and Procedure

The stimuli, the apparatus and the procedure in Experiment 4 were the same as in Experiment 3. The only difference between those two experiments was how participants were assigned to conditions. Like in Experiment 1 and Experiment 2, we had a full within-participant design in Experiment 3 as well. Therefore, each participant completed each condition. In Experiment 4 each participant completed only one condition, either the no-movement or the stop-and-go condition.

### Results

Mean embodiment ratings are plotted as a function of rating position (1^st^–9^th^) and condition (no-movement vs. stop-and-go condition) in Figure 5(b).

#### Embodiment Phase

We performed a 2 × 2 ANOVA with the within-subject factor rating position (1^st^ vs. 4^th^ rating position) and the between-subjects factor condition (no-movement vs. stop-and-go condition). There was a main effect of rating position, *F* (1, 114) = 104.58, *p* < .001, η_p_^2^ = .48. Neither the main effect of condition, *F* (1, 114) < 1, nor the interaction was significant, *F* (1, 114) < 1. Although the result of a Bayesian *t* test did not support the null hypothesis that ratings are equal across conditions for the average embodiment ratings across all rating positions of the embodiment phase, BF_01_ = 0.87. However, the result of a further Bayesian *t* test suggested that rating levels at rating position 4 were comparable between conditions, BF_01_ = 3.97.

#### Disembodiment Phase

We computed a 2 × 2 ANOVA with the within-subject factor rating position (5^th^ vs. 7^th^ rating) and the between-subjects factor condition (no-movement vs. stop-and-go condition). The analysis revealed a significant main effect of rating position *F* (1, 114) = 29.95, *p* < .001, η_p_^2^ = .21, while the main effect of condition, *F* (1, 114) < 1, and the interaction, F (1, 114) < 1, were not significant. Follow-up *t*-tests contrasting the 5^th^ and 6^th^ rating position for each condition revealed significant effects for the no-movement condition, *t* (55) = 3.92, *p* < .001, *d*_*z*_ = 0.52, and the stop-and-go condition, *t* (59) = 4.60, *p* < .001, *d*_*z*_ = 0.59. Additional *t*-tests contrasting the 6^th^ and 7^th^ rating position revealed no significant effects for neither condition (no-movement condition: *t* (55) = −0.28, *p* = .784, *d*_*z*_ = −0.04; stop-and-go condition: *t* (59) = 1.22, *p* = .226, *d*_*z*_ = 0.16). A Bayesian *t* test comparing embodiment ratings between the 6^th^ and 9^th^ rating position for the no-movement condition suggested that ratings were comparably high at both rating positions, BF_01_ = 4.84. Further we tested whether embodiment was fully reinstated in the stop-and-go condition after participants started moving again by comparing the 5^th^ and 8^th^ rating as well as the 5^th^ and 9^th^ rating with Bayesian *t*-tests respectively. The results of Bayesian *t*-tests comparing the 5^th^ and 8^th^ rating and the 5^th^ and 9^th^ rating, respectively, suggested for rating position 8, BF_01_ = 5.14, and also for rating position 9, BF_01_ = 4.37 that these ratings were comparably high to the level of the last rating of the embodiment phase (rating position 5). As in the preceding experiments, we also checked for Experiment 4 whether participants engaged in the attention task properly. Performance during the attention task was correct in 88.09% of trials.

Note that the data analysis for the disembodiment phase slightly deviated from the preregistered plan. Preregistered *t*-tests between conditions for rating position 6 and for rating position 7 were not performed because in contrast to our prediction, descriptively, there was no evidence in the current data to assume a difference between both conditions for the 6^th^ or 7^th^ rating position (see Figure 5(b)).

### Discussion

In both conditions we observed that embodiment for the virtual hand gradually increased during the embodiment phase and rapidly decreased as soon as participants stopped moving. In the no-movement condition, after the initial fast disembodiment of the virtual hand, ratings remained at this lower level until the end of the disembodiment phase (6^th^ rating–9^th^ rating). In the stop-and-go condition, ratings rapidly increased during the second part of the disembodiment phase after participants resumed moving. However, like in Experiment 3, we again observed a comparable decrease of embodiment ratings during the first part of the disembodiment phase for both conditions regardless of different task instructions in each condition. Although the reinstatement of embodiment after participants resumed moving in the disembodiment phase of the stop-and-go condition was descriptively faster in Experiment 3 than in Experiment 4, the overall dynamic was comparable to the increase of embodiment ratings during the initial part of the embodiment phase. In sum, the results of Experiment 3 and Experiment 4 do not support the hypothesis that the observed fast disembodiment might reflect a preparedness to disembody the previously embodied object due to an instruction-based anticipation of the requirements of a future task.

## General Discussion

The present web-based study comprises four experiments to explore the temporal dynamics of disembodiment of a previously embodied 2D virtual hand that could be controlled through movements of a computer mouse or on a touchpad. In all experiments, participants first moved the virtual hand continuously back-and-forth from one side of the computer screen to the other. This procedure aimed at inducing a sense of embodiment for the virtual hand and was therefore referred to as embodiment phase. During the subsequent disembodiment phase, we investigated how fast the previously embodied virtual hand would become disembodied after participants stopped moving (Experiment 1 and Experiment 2). Moreover, we assessed whether the temporal dynamics of disembodiment can be modified through anticipation of upcoming task demands based on instruction (Experiment 3 and Experiment 4). We found that sense of embodiment for the virtual hand decreased rapidly during the initial part of the disembodiment phase. However, in contrast to prior work, the better temporal resolution of the present study allowed to reveal that ratings did not drop immediately after stopping to move but decreased more gradually (Eck et al., 2022). This was the case across experiments, and we did not find evidence for additional modulatory effects of instruction on the dynamics of disembodiment. The results suggest that an actively controlled virtual object becomes embodied due to integration of incoming correlated visual signals, resulting from movements of the virtual object, and motor signals, resulting from one’s own corresponding movements. However, this virtual body extension becomes rapidly disembodied if the body representation is no longer updated through incoming correlated sensorimotor signals relating to the virtual hand and the real hand. The observation of a gradual pattern for the increase of ratings during the embodiment phase and a comparable pattern, although faster, for the decrease of ratings during the initial part of the disembodiment phase possibly imply the involvement of multisensory integration mechanisms in both expanding and reducing of the body representation. The importance of sensorimotor experience for updating the body representation was further highlighted by the finding that anticipating immediate reuse of the virtual object based on current task demands is not sufficient to prevent disembodiment.

### Fast But Not Instant Disembodiment

The present study used refined methods of previous work (Eck et al., 2022). In this earlier study we had observed a seemingly instant drop of ratings to the level of preembodiment right after participants stopped moving. The observed instant drop of embodiment ratings was unexpected as we had predicted a gradual decrease of embodiment ratings. This prediction was based on findings from studies which investigated the disembodiment of previously embodied physical rubber hands (Abdulkarim et al., 2021; Pfister et al., 2021). We hypothesized that the instant disembodiment that we observed in the previous study might have been an artefact of the relatively long duration of the intervals (20 seconds) between ratings. The results of the present experiments lend strong support to this hypothesis by using increased temporal resolution to uncover that the disembodiment of a previously embodied virtual object unfolds more gradually following a curved decay function with a rapid initial decrease.

The finding of a more gradual pattern of disembodiment in the current study, is consistent with previous studies that observed gradual disembodiment for physical rubber hands (Abdulkarim et al., 2021; Pfister et al., 2021). However, in these studies, disembodiment of a rubber hand still was clearly slower than the observed disembodiment of the 2D virtual hand in the current study. This difference might be explained within the causal inference framework that suggests that the cognitive system infers based on accumulating evidence from present sensory experience and prior knowledge what is part of one’s own body and what is not (de Vignemont, 2010; Ernst & Bülthoff, 2004; Samad et al., 2015). In a rubber hand illusion, the experience of multisensory signals coming synchronously from the rubber hand and the covered real hand and prior knowledge (anatomically plausible orientation and appearance of the rubber hand) provide strong evidence for the cognitive system to infer that the rubber hand is a part of one’s own body (Samad et al., 2015; Tsakiris et al., 2010). However, embodiment of anatomically less plausible physical or virtual objects is mainly based on the experience of agency resulting from actively controlling the object (Farnè et al., 2005; Ma & Hommel, 2015a, 2015b; Short & Ward, 2009). Therefore, the relatively slower disembodiment that was observed after a rubber hand illusion possibly reflects that embodiment for the rubber hand might have been maintained to some degree even after multisensory stimulation stopped. In contrast, the relatively faster disembodiment of the 2D virtual hand in the current study, possibly suggests that there was no further updating of the body representation at all after participants stopped moving because initial embodiment was mainly based on the experience of a match between one’s own active movements and movements of the virtual hand. It would be interesting to test this hypothesis in future studies through comparing the temporal dynamics of disembodiment of a previously embodied virtual object between different contexts of HCI that vary with respect to the degree of immersion (Bergström et al., 2019; Maselli & Slater, 2013). According to the causal inference model, we would expect a faster disembodiment of virtual body extensions for nonimmersive than for immersive HCI.

### Actual Experience versus Anticipation of Control

Several studies suggest that emergence of a sense of embodiment for objects that have little or no anatomical plausibility might not necessarily depend on agency that is currently experienced for the object (Bassolino et al., 2010; Cardinali et al., 2021; Liepelt et al., 2017; Serino et al., 2007). According to this evidence, sense of embodiment for external objects might also emerge based on agency experience that is remembered from past interactions with the object or based on agency experience that is imagined for potential interactions with the object in the future. Based on these, in Experiment 3 and 4 we investigated effects of agency experience representations on disembodiment. For this we tested whether the decrease of ratings after stopping to move would be less pronounced if explicitly telling participants that they will have to actively control the virtual hand again after a brief movement break (stop-and-go-condition) as compared to a condition where participants were only told to stop moving (no-movement condition). However, we found that ratings in the stop-and-go condition decreased as fast as in the no-movement condition. This finding suggests that actual experience of agency is crucial for inducing and maintaining a sense of embodiment for a virtual object and cannot be substituted by anticipation of agency experience.

This finding is at odds with another study that investigated embodiment of a mouse cursor (Bassolino et al., 2010). In this study, a mouse cursor was not only embodied after a period of actively controlling it through moving the computer mouse (active condition) but also when holding the computer mouse passively (passive condition; Bassolino et al., 2010). While in the active condition embodiment of the mouse cursor was observed independent of whether the computer mouse was operated with the right or the left hand, in the passive condition, embodiment of the mouse cursor occurred only after holding the computer mouse with the right hand. The observed difference between hands for the passive condition, possibly suggest that embodiment of the mouse cursor was mainly based on retrieval of previous agency experience for the mouse cursor because all participants reported an extended history of using the computer mouse with the right but not the left hand (Bassolino et al., 2010). Thus, while in the Bassolino et al. (2010) study a virtual object was embodied even without current experience of active control, results of the current study suggest that current sensorimotor experience is necessary to maintain embodiment of a virtual object.

However, it is difficult to directly compare the current findings with the findings from the Bassolino et al. (2010) study for two reasons. First, the focus of both studies differed. While the study form Bassolino and colleagues focused on embodiment of a virtual object the current study investigated prevention of disembodiment of a previously embodied virtual object. Accordingly, in the current study, the passive phase always came directly after a phase of ongoing active experience of agency. Hence, the experience of stopping to move was closely related to the preceding active experience. There was no such interaction between the active and passive condition in the Bassolino et al. (2010) study because both conditions were independent of each other. Second, both studies used different measures of embodiment. While in the current study sense of embodiment for the virtual object was reflected through increase of subjective ratings of body ownership experience, in the Bassolino et al. (2010) study embodiment for the virtual object was inferred from performance in a version of the cross-modal congruency task, reflecting changes in the self-location component of embodiment (Kilteni et al., 2012a). Possibly, mere representation of agency and actually experienced agency, might affect different components of embodiment respectively. Further, studies showing discrepancies between implicit and explicit measures of embodiment (Ma et al., 2021; Riemer et al., 2019; Rohde et al., 2011), possibly suggest that effects of remembered or anticipated agency on the embodiment or disembodiment of external objects might only become evident with implicit but not with explicit measures. Clearly, more studies are needed to better understand the effects of agency experience and agency representation on embodiment and disembodiment.

### Limitations and Transfer

Figure 3 shows that in Experiment 1 and 2, descriptively, ratings right at the beginning of the embodiment phase were higher in the no-movement condition than in the active condition. This descriptive observation possibly suggests that relatively longer overall durations of active control experience in the active condition compared to the no-movement condition might have resulted in carry-over effects from one condition to the other due to the trial-by-trial alternation of conditions. At the beginning of trials in the active condition, the sense of embodiment for the virtual hand that was induced during the embodiment phase of preceding no-movement condition trials was completely reversed due to relatively long experience of no sensorimotor updating during the disembodiment phase and the break between trials. In contrast, prior to trials in the no-movement condition a lack of sensorimotor updating was only experienced during the short break between trials. This interval might have been not enough time for a full reverse of the previously induced sense of embodiment for the virtual hand. This possible methodological limitation should be ruled out in future studies through implementation of longer breaks in-between trials. Nevertheless, besides the length of the breaks another factor might possibly explain the descriptively observed carry-over effects. We instructed participants for the breaks to take the hand off the computer mouse or touchpad and to move it around because we assumed that this procedure should induce rapid disembodiment of the previously embodied virtual hand. However, participants might have refrained from doing this.

The current study was a web-based study. Therefore, we cannot rule out that participants actually followed all instructions, as participants ran the study remotely using their own equipment and we neither observed them live through video-conference tools nor videotaped the respective experimental sessions. However, there are at least two reasons to assume that most of the participants were compliant with the instructions. First, several studies showed that compliance and trustworthiness of participants in studies that were conducted online is comparably high to studies that were conducted in the lab (Germine et al., 2012; Rodd et al., 2024). Second, the general pattern of results, that embodiment for a virtual object gradually increased when it was actively controlled through movements of a computer mouse or on a touchpad and rapidly decreased when controlling of the virtual object was no longer required, were replicated across four experiments. Further, these observations are also consistent with findings from a previous web-based study that relied on a similar methodological setup (Eck et al., 2022). Repeated replication of these findings would be highly unlikely if most of the participants did not follow the instructions.

Despite the relative robustness of the current findings as suggested by the repeated replication, an important question is, whether the current findings also have relevance for practical application in addition to their theoretical implications for models of embodiment in the context of virtual interactions. Applied fields from teleoperation to video gaming cover a broad range of virtual interactions, including fully immersive virtual reality technology (Baceviciute et al., 2022; Dincelli & Yayla, 2022; Moniruzzaman et al., 2022; Morimoto et al., 2022). This poses the question of whether our results generalize across different fields, and how they scale with future technological advances. Evidence from studies that investigated (dis)embodiment in more immersive HCI settings as compared to our setting provides a highly similar pattern of results so that the basic principles observed in the present data should indeed be expected to scale up (Bergström et al., 2019; Brugada-Ramentol et al., 2019; D’Angelo et al., 2018; Kilteni et al., 2012b; Kocur et al., 2022; Kokkinara & Slater, 2014; Ma & Hommel, 2015a, 2015b; Sanchez-Vives et al., 2010; Short & Ward, 2009). That is: While embodiment of a virtual object might be easier to induce in more immersive HCI settings (Maselli & Slater, 2013), the emerging sense of embodiment for the virtual object still rests on the same principles of multisensory integration that are also supposed to drive embodiment of a 2D virtual hand in the present setup (Bergström et al., 2019; Kilteni et al., 2015; Noel et al., 2018; Sengül et al., 2012). It, therefore, seems warranted to expect generalization (Pfister, 2022), especially for nonimmersive HCI applications that involve direct control of a virtual object. This calls for a thorough re-evaluation of current interventions, particularly if an intervention aims at instilling longer-lasting changes.

More specifically, the current results might be applied to the area of serious video games, that have in recent years become an important tool for neuromotor rehabilitation (Massetti et al., 2018; Zanatta et al., 2022). For example, a recent study found that patients suffering from multiple sclerosis showed better improvement of gross and fine manual dexterity after an intervention that combined a conventional neuromotor rehabilitation program with serious video game playing sessions as compared to a group of patients that received only the conventional rehabilitation program (Cuesta-Gómez et al., 2020). In this study, participants controlled schematic virtual hands through movements of their real hands and translation of real to virtual movements was managed by a motion tracker. Controlling of virtual hands in video game-based HCI settings through movements of one’s own real hands was also the basic procedure in other studies on motor rehabilitation programs (Lee et al., 2021; van Beek et al., 2019). It has been argued that for successful neuromotor rehabilitation serious video games must be motivating as well as easy to learn and to use (Zanatta et al., 2022). Virtual interactions are assumed to be experienced as more fluent and therefore also more engaging if the virtual object that represents the intentions of the user during the current virtual interaction is embodied (e.g., mouse cursor, virtual arm, or avatar; Haans & IJsselsteijn, 2012; Seinfeld et al., 2021). The current finding that agency-based embodiment of controllable virtual objects need some time to emerge suggest that pretrainings aiming at inducing embodiment for the critical virtual object before starting the actual serious video game-based intervention might enhance performance and learning during the intervention. The current observation of a rapid disembodiment after stopping to actively control the virtual object, in turn, suggests that when changing tasks undesirable carry-over effects form one virtual interaction to another are unlikely. However, fast disembodiment also implies that desirable transfer effects from the digital environment of the video game to relevant everyday life tasks might be poor. Combining interventions based on serious video games with exercises based on interactions with physical objects might possibly enhance such transfer effects. Thus, interventions that combine conventional neuromotor rehabilitation programs with serious video games-based trainings might be not only superior to conventional programs alone (Cuesta-Gómez et al., 2020) but also preferable to potential interventions that solely rely on playing of serious video games.

### Conclusion

The results of the current study show that a simple virtual object that is controlled trough active movements of an input device and moves congruently with those movements can be experienced as a part of one’s own body. However, this sense of embodiment for the virtual object rapidly decreases if sensorimotor updating of the current body representation stops, that is, when controlling of the virtual object is no longer relevant for a current task. Disembodiment cannot be prevented or decelerated through expectations of upcoming interactions with the same object in the immediate future. These findings suggest that current experience of active control over a virtual object is not only crucial for inducing but also for maintaining embodiment. The observed dynamics of disembodiment pose profound challenges to attempts of instilling longer-lasting changes of the body representation during rehabilitation, and they rather support applications that aim at minimizing aftereffects of embodiment, for example, during teleoperation.

## Key Points

• Virtual objects that are controlled through movements of the user can become embodied based on experience of active control over the object.
• Interruptions of active control experience result in rapid disembodiment of the previously embodied virtual object.
• Anticipating repeated upcoming interactions for an imminent task cannot prevent disembodiment during interruptions of control experience.
• The findings suggest a flexible adaptation of the body representation to interactions in digital environments which is based on current sensorimotor experience.
